# Understanding G Protein Selectivity of Muscarinic Acetylcholine Receptors Using Computational Methods

**DOI:** 10.3390/ijms20215290

**Published:** 2019-10-24

**Authors:** Luis Jaimes Santiago, Ravinder Abrol

**Affiliations:** Department of Chemistry and Biochemistry, California State University, Northridge, CA 91330, USA

**Keywords:** receptor–G protein interactions, molecular mechanics/Poisson–Boltzmann surface area (MMPBSA), allostery, membrane protein simulations, GPCR activation, molecular dynamics

## Abstract

The neurotransmitter molecule acetylcholine is capable of activating five muscarinic acetylcholine receptors, M1 through M5, which belong to the superfamily of G-protein-coupled receptors (GPCRs). These five receptors share high sequence and structure homology; however, the M1, M3, and M5 receptor subtypes signal preferentially through the Gαq/11 subset of G proteins, whereas the M2 and M4 receptor subtypes signal through the Gαi/o subset of G proteins, resulting in very different intracellular signaling cascades and physiological effects. The structural basis for this innate ability of the M1/M3/M5 set of receptors and the highly homologous M2/M4 set of receptors to couple to different G proteins is poorly understood. In this study, we used molecular dynamics (MD) simulations coupled with thermodynamic analyses of M1 and M2 receptors coupled to both Gαi and Gαq to understand the structural basis of the M1 receptor’s preference for the Gαq protein and the M2 receptor’s preference for the Gαi protein. The MD studies showed that the M1 and M2 receptors can couple to both Gα proteins such that the M1 receptor engages with the two Gα proteins in slightly different orientations and the M2 receptor engages with the two Gα proteins in the same orientation. Thermodynamic studies of the free energy of binding of the receptors to the Gα proteins showed that the M1 and M2 receptors bind more strongly to their cognate Gα proteins compared to their non-cognate ones, which is in line with previous experimental studies on the M3 receptor. A detailed analysis of receptor–G protein interactions showed some cognate-complex-specific interactions for the M2:Gαi complex; however, G protein selectivity determinants are spread over a large overlapping subset of residues. Conserved interaction between transmembrane helices 5 and 6 far away from the G-protein-binding receptor interface was found only in the two cognate complexes and not in the non-cognate complexes. An analysis of residues implicated previously in G protein selectivity, in light of the cognate and non-cognate structures, shaded a more nuanced role of those residues in affecting G protein selectivity. The simulation of both cognate and non-cognate receptor–G protein complexes fills a structural gap due to difficulties in determining non-cognate complex structures and provides an enhanced framework to probe the mechanisms of G protein selectivity exhibited by most GPCRs.

## 1. Introduction

G-protein-coupled receptors (GPCRs) comprise the largest superfamily of integral membrane proteins, covering ~3% of the human proteome. They mediate pleiotropic transmembrane (TM) signal transduction by allosterically facilitating information transfer across the cellular membrane in response to extracellular signals [1,2] and converting them into one or more intracellular signaling cascades [3]. The extracellular signals range from photons (for rhodopsin) and small molecules (like neurotransmitters, metabolites, odorants, tastants, etc.) to large oligopeptides (like chemokines, incretins, etc.). This critical role of GPCRs in cellular signaling makes these receptors therapeutic targets in a large number of diseases (with ~50% of all modern drugs targeting GPCRs [4]), either due to their direct role in the pathophysiology of a specific disease or due to their ability to modulate a set of signaling cascades implicated in a disease [5]. For example, non-synonymous single-nucleotide polymorphisms (nsSNPs) in GPCRs that are linked with specific disease pathologies can cause pathophysiological changes due to altered signaling via conformational changes in the receptor.

Signal transduction by GPCRs starts with an agonist ligand (like a neurotransmitter acetylcholine) binding to the receptor and activating it, which in turn activates one of the G proteins. After G protein activation, GPCR receptor kinases (GRKs) and arrestins are recruited for receptor desensitization and endocytosis, which can recycle the receptor back to the plasma membrane for the next round of signaling. The GRKs and arrestins can trigger their own signaling cascades [6,7], which is referred to as G-protein-independent signaling. In this context of ligand-induced GPCR signaling cascades [8], the receptor and the ligand play unique functional roles.

Each receptor has evolved to activate mainly one subfamily of G proteins. Since each G protein subfamily triggers a specific set of signaling cascades inside the cells, a receptor’s natural specificity for a G protein subfamily allows it to activate only a specific set of signaling cascades downstream of that G protein subfamily. Some receptors are known to couple to a second G protein subfamily, but those couplings are usually weak. As mentioned above, a receptor can additionally signal through arrestins and GRKs, which means that the activation of a single receptor by an agonist can cause multiple pathways to be activated. This is called balanced signaling and can cause GPCR-targeted drugs to have on-target side effects. For example, the drug candidate niacin activates its receptor GPR109A and causes beneficial anti-lipolytic effects through G protein signaling triggered by that receptor; however, niacin also causes a major side effect of cutaneous flushing through arrestin-mediated signaling triggered by the same receptor [9]. Such observations are generating novel therapeutic approaches utilizing biased ligands aimed at minimizing on-target side effects through biased signaling [10,11,12].

**Figure 1:**
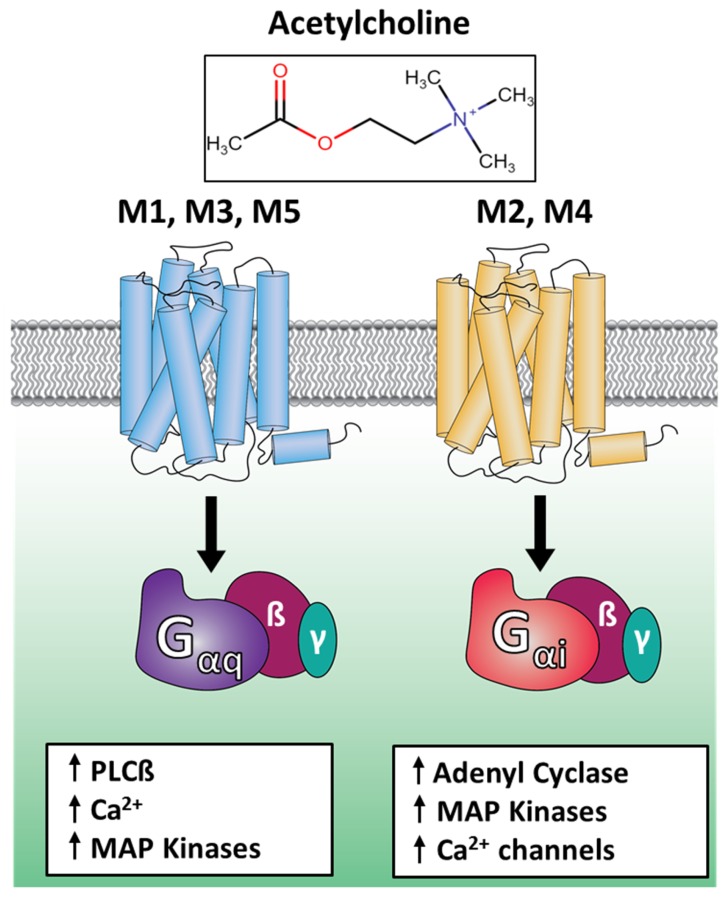
G protein selectivity and signaling differences in the muscarinic receptor family.

A ligand like acetylcholine can activate multiple receptor subtypes. For example, acetylcholine activates the five muscarinic receptor subtypes M1 through M5, where each of the receptor subtypes activates one specific subfamily of G proteins (Figure 1) [13]. The receptor subtypes M1, M3, and M5 couple to the Gq/11 subfamily of G proteins, which are responsible for the activation of phospholipase C-β (PLC-β) that results in the production of signaling molecules inositol triphosphate (IP3) and diacylglycerol (DAG). The M2 and M4 receptor subtypes couple to the Gi/o subfamily of G proteins, which cause a decrease in the production of second messenger molecule cAMP, responsible for a different set of cellular and physiological responses. M2 has been shown to couple weakly to Gs and Gq proteins as well [14,15]. So, a single ligand like acetylcholine can cast a wider net of signaling cascades than is possible with the activation of one receptor alone; this is because this ligand can activate multiple receptor subtypes that have each evolved to couple to a specific subfamily of G proteins. This work is aimed at gaining a mechanistic understanding of this specificity of receptor–G protein interactions (Figure 1) in the muscarinic receptor family because this family is the most homologous compared to any other receptor family and because if there are any clear structural determinants of G protein selectivity, they should be easy to identify for this family. This knowledge will also be critical to designing drugs that need to target only one specific receptor–G protein interaction for therapeutic benefit. As an example, targeting the M2 receptor with an agonist drug can cause side effects coming from its weak coupling to the Gs and Gq proteins or even from the M1 receptor, so mechanistic understanding of G protein interactions with M1 and M2 receptors can help us to rationally design drugs that only activate the Gi/o proteins through the M2 receptors and not the other G proteins or the M1 receptor, to minimize unwanted side effects.

Some studies have looked at this innate G protein specificity of receptors using structural bioinformatics [16], genomics [17], and machine learning [18] approaches. One of these studies [17] identified individual residues as potential selectivity determinants; however, it did not provide the structural mechanisms behind the ligand-induced innate G protein specificity. In addition, the sequence-level and structure-level associations do not capture the biophysical complexity of the physical interaction between the receptor and the G proteins, which is very dynamical in nature. This is supported by an ever-increasing number of experimental and computational studies [8,19,20,21,22,23,24] that point to different ensembles of receptor conformations behind the signaling of GPCRs. Such mechanistic studies are getting a major boost due to the recent dramatic increase in available GPCR structures. G protein specificity can also be controlled by regulating gene expression such that the undesirable G protein is not expressed in a specific cell; however, this has not been found to be the case in general [17].

Experimental structures are now available for ~60+ GPCRs with a wide coverage of sequence space, ~40+ of which are for human sequences covering all GPCR phylogenetic families. Some receptors have been crystallized in their active conformations, capable of coupling to G proteins and arrestins. Human β2 adrenergic receptor was crystallized in an active conformation in complex with the full heterotrimeric Gs protein, providing a snapshot of the conformational changes in both the receptor and the Gs protein during GPCR activation [25,26]. The adenosine A1 receptor complex with the full Gi2 protein [27], the muscarinic M1 receptor complex with G11, and the M2 receptor complex with Go have been obtained [28] with the increasingly popular cryo-EM approach [29]. All these structures are catalyzing the full spectrum of mechanistic studies into GPCR biology to understand their functional and pharmacological implications [30,31,32]. A recent review of all these G-protein-bound receptor structures [33] found no clear determinants of G protein selectivity through a comparison of available Gi/o-, Gq-, and Gs-bound structures. In addition, a computational study of only the α5 helix of Gi/o, Gq, and Gs proteins interacting with cognate and non-cognate receptors [34] demonstrated the tunable conformational malleability of the intracellular-facing region of GPCRs that can allow the receptors to accommodate multiple G proteins or only one.

The recently described structures of the cognate M1:G11 and M2:Go complexes [28] have provided a framework to think about the potential selectivity determinants for observed couplings between receptors activated by the same ligand (acetylcholine in this case) and their preferred G protein partners. These structures show that the G protein orients differently in the cognate receptor–G protein complexes. In the absence of the structures of non-cognate complexes (M1:Go complex and M2:G11), it is not clear how great a role is played by the observed G protein orientation differences in the cognate complexes for determining the G protein preferences because not all binding orientations would be conducive to G protein activation. The non-cognate complexes are expected to be less stable due to weak coupling between the receptor and the G protein and, hence, difficult to characterize with structure determination efforts. The M1 receptor in the M1:G11 complex shows more extensive interactions with the G protein compared to the M2 receptor in the M2:Go complex. This also paints an incomplete picture of G protein selectivity determinants because based on these cognate complex structures alone, one cannot point to specific sequence and structural characteristics of the receptors that result in their G protein preferences. The structures of non-cognate complexes (M1:Go and M2:G11) can begin to provide a more complete understanding of G protein selectivities; however, they may be difficult to obtain experimentally.

Structural modeling of these non-cognate complexes in the membrane environment combined with the structural modeling of cognate complexes can complement and supplement the understanding gained from the cryo-EM structures [28]. The biophysical interaction between a receptor and its preferred G protein will be governed by two factors: thermodynamics and kinetics. Thermodynamics captures the energetics of association between the two proteins and kinetics governs how fast these two proteins associate and dissociate. A recent study has shown that the muscarinic M3 receptor’s interaction with Gq and Go proteins is governed by the affinity of the interaction (thermodynamics) as opposed to the on-rate of the interaction (kinetics) [35].

In this study, we used computational biophysical methods and the cryo-EM structures of M1:G11 and M2:Go complexes [28] as templates to generate the structures of cognate (M1:Gαq and M2:Gαi) and non-cognate (M1:Gαi and M2:Gαq) complexes of M1 and M2 receptors with Gi and Gq proteins. The structures of these four complexes were relaxed in the lipid bilayer environment using molecular dynamics (MD) simulations. The resulting ensembles of conformations of all four complexes were then used to analyze the structural similarities and differences and the thermodynamics of receptor interactions with G proteins to understand the mechanisms behind that selectivity. This was done through the membrane adaptation of the MMPBSA (molecular mechanics/Poisson–Boltzmann surface area) approach [36,37], which captures the free energy of binding between two biomolecules.

In the next section, the results of the MD simulations of all four receptor–G protein complexes are analyzed in terms of receptor structures, G protein orientations, receptor–G protein binding free energies, receptor–G protein noncovalent interactions, and intra-receptor interactions to understand the basis of G protein selectivity displayed by the M1 and M2 receptors. The structures of the cognate and non-cognate complexes are also used to understand the role of known receptor residues that have been implicated before in G protein couplings and/or selectivity. This is followed by a section on materials and methods that describes how the structures were built, how the MD simulations were carried out, how the free energies were calculated, and how the structures were analyzed.

## 2. Results and Discussion

### 2.1. Structural Modeling and Generation of Receptor–G Protein Conformational Ensembles

**Figure 2:**
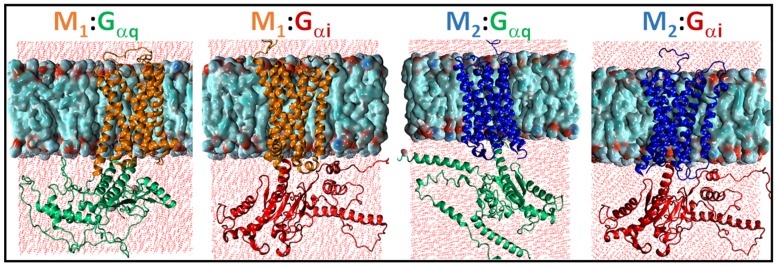
The four receptor–G protein complexes embedded in the membrane environment. The M1 receptor is shown in orange. The M2 receptor is shown in blue. The Gαq protein is shown in green and the Gαi protein is shown in red. The lipids are shown in space-filling representation and the two layers of water molecules above/below the lipid bilayer are shown in red.

The simulation systems for the two cognate receptor–G protein complexes (M1:Gαq and M2:Gαi) and the two non-cognate complexes (M1:Gαi and M2:Gαq) in the lipid bilayer environment are shown in Figure 2 below.

Section 3 describes how these four complex systems were built and then relaxed in their native environment using a series of energy minimization and molecular dynamics (MD) steps. Each of the four complexes was relaxed for 2 μs by running four parallel MD simulations of 500 ns duration each. The resulting snapshots of the four systems were saved for structural and thermodynamic analysis. Based on the root-mean-squared deviation (RMSD) of the receptor backbone atoms (Supplementary Figures S1–S4) in each simulation copy, we used the system conformations from last 400 ns from each copy for further analysis, resulting in molecular coordinates covering effectively 1.6 μs of MD simulations for each complex. An average conformation representing this 1.6 μs of simulation time was obtained for each complex based on the procedure described in Section 3.

### 2.2. Comparison of MD Structures with cryo-EM Structures

**Figure 3:**
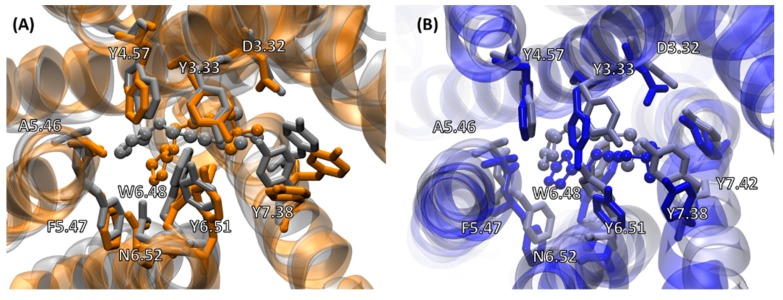
(**A**) Overlay of ligand binding sites in the M1:Gαq (orange) and M1:G11 (gray) structures; (**B**) Overlay of the M2:Gαi (blue) and M2:Go (blue-gray) structures. The receptor side chains are shown in licorice (solid bonds) representation, and the ligand is shown in ball-and-stick representation. The G proteins are not shown as they do not interact with the ligands.

Figure 3 shows an overlay of the average structures from MD simulations for the cognate complexes (M1:Gαq and M2:Gαi) with the corresponding cryo-EM structures (M1:G11 and M2:Go). The focus here is on the agonist iperoxo’s binding site and interacting residues. The RMSD of the ligand’s heavy atoms in the average structure of the M1:Gαq complex relative to the M1:G11 cryo-EM complex was 0.946 Å. The ligand RMSD in the average structure of the M2:Gαi complex relative to the M2:Go cryo-EM complex was 1.701 Å. The ligand in M2:Gαi was shifted slightly away from transmembrane helix 3 (TM3) compared to its position in the M2:Go structure, which could be due to minor adjustments in the M2 conformation when bound to Gαi. The seven TM helix domains for the two receptors referred to in this study are given in Supplementary Table S0. The sidechains of the interacting residues occupy similar positions around the ligand pocket in the MD simulations as in the cryo-EM structures. So, the simulations captured the agonist position and interactions with the receptors appropriately.

**Figure 4:**
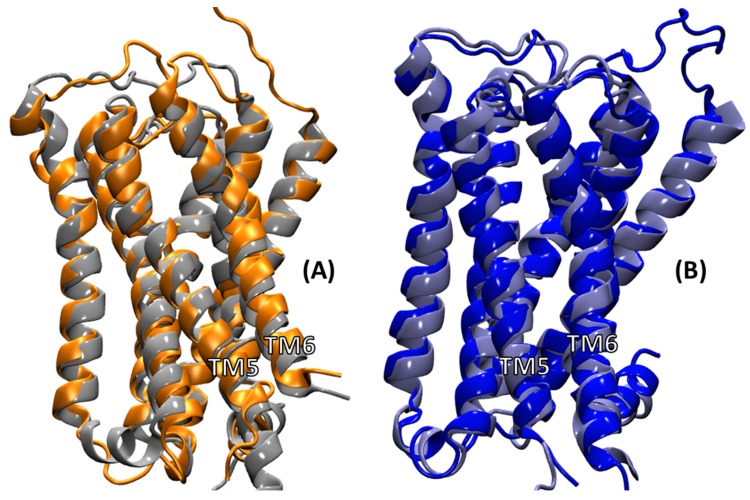
Overlay of the M1 and M2 receptors for the cognate complexes with the corresponding cryo-EM complexes. (**A**) Overlay of M1 (orange) from the average M1:Gαq molecular dynamics (MD) complex and M1 (gray) from the M1:G11 cryo-EM complex; (**B**) Overlay of M2 (blue) from the average M2:Gαi MD complex and M2 (gray) from the M2:Go cryo-EM complex.

At the receptor level, Figure 4 shows an overlay of the average receptor structures from the MD simulations of cognate complexes with their corresponding cryo-EM counterparts. The TM domains showed good overlap for both the M1 and M2 conformations when compared to their conformations observed in the cryo-EM structures. The RMSD of the TM domain of the M1 receptor from M1:Gαq complex relative to the M1 receptor from the M1:G11 complex was 2.34 Å. The corresponding number for the TM domain of the M2 receptor from the M2:Gαi complex relative to the M2 receptor from the M2:Go complex was 2.12 Å. The loop regions do show variations, which is expected as the loops are usually quite flexible, and the cryo-EM conditions captured only one of the possible loop conformations. The TM5 domain of the M1 receptor was longer than that for the M2 receptor in the cryo-EM structure.

The longer TM5 helical extension in M1 compared to in M2 observed in the cryo-EM structures unraveled slightly at the bottom of TM5 during the MD simulations; however, it preserved the extensive contacts with the G protein in the M1 complexes, as will be discussed later. The helical structure of the intracellular loop (ICL2) observed in the cryo-EM structure of M1 unraveled a bit during the MD simulation (Figure 4A); however, the helical ICL2 observed in the cryo-EM structure of M2 was maintained during the MD simulation (Figure 4B).

**Figure 5:**
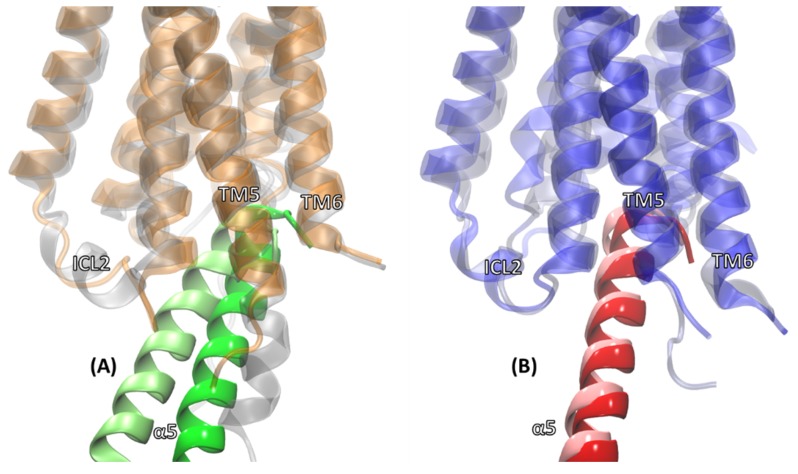
Overlay of the α5 helices of G proteins from the cognate complexes with the corresponding cryo-EM complexes. (**A**) Overlay of the Gαq α5 helix (dark green) from the average M1:Gαq MD complex and the G11 α5 helix (light green) from the M1:G11 cryo-EM complex; (**B**) Overlay of the Gαi α5 helix (red) from the average M2:Gαi MD complex and the Go α5 helix (pink) from the M2:Go cryo-EM complex. The rest of the Gα protein is hidden for clarity.

Next, looking at the G protein part of the complexes, Figure 5 shows an overlay of the α5 helix of the G protein from MD simulations with that of the G proteins from the cryo-EM structures. Figure 5A shows the α5 helix of the Gαq protein (dark green) from the cognate M1:Gαq complex compared to the α5 helix of the G11 protein (light green) from the M1:G11 cryo-EM complex. At first, they look like slightly different orientations of the α5 helix relative to the receptor; however, this difference could be due to fact that MD simulations used the Gαq protein and the cryo-EM structure is with the G11 protein. In addition, this different orientation is in line with the variation seen in the X-ray and cryo-EM structures [33,34] of the same G proteins (see, for example, Table S2 in ref [34] for different orientation angles for the same G protein, which shows that the orientation angles are different by ~10–15 degrees for the same G protein, probably capturing the dynamic nature of the receptor–G protein interactions). Figure 5B shows the α5 helix of the Gαi protein (red) from the cognate M2:Gαi complex compared to the α5 helix of the Go protein (pink) from the M2:Go cryo-EM complex. The two α5 helices (Gαi and Go) show a very similar orientation relative to the M2 receptor, which means that M2 engages with the two proteins in the same way. A comparison of the α5 helix orientations in cognate M1 and M2 complexes from MD simulations (Figure 5) with the respective cryo-EM complexes suggests that the M1 receptor can engage with G proteins in multiple orientations, unlike the M2 receptor, which engages in one main orientation. This has interesting implications for cognate vs non-cognate interactions, which can affect whether a given receptor–G protein coupling orientation will lead to G protein activation or not. This is still an open question that remains to be looked at and is beyond the scope of this study.

### 2.3. Structural Comparison of Cognate and Non-Cognate Complexes

**Figure 6:**
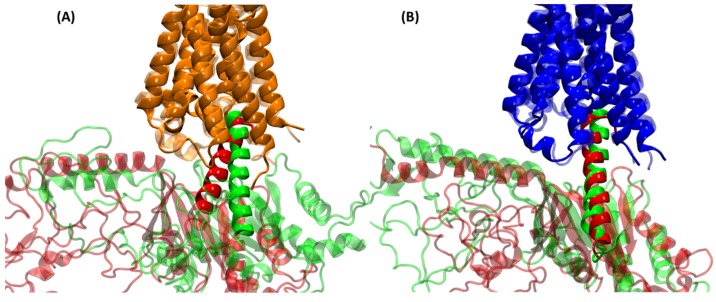
Comparison of the cognate complexes with the non-cognate complexes. (**A**) Overlay of M1:Gαq (orange/green) and M1:Gαi (light orange/red) complexes from the average MD structures; (**B**) Overlay of M2:Gαq (blue/green) and M2:Gαi (light blue/red) complexes from the average MD structures.

In order to better understand the selectivity of the cognate receptor–G protein interactions, it is useful to overlay the cognate and non-cognate G protein partners bound to the same receptor. Figure 6 shows the average MD structures of the M1 and M2 receptors bound to Gαi and Gαq.

**Figure 7:**
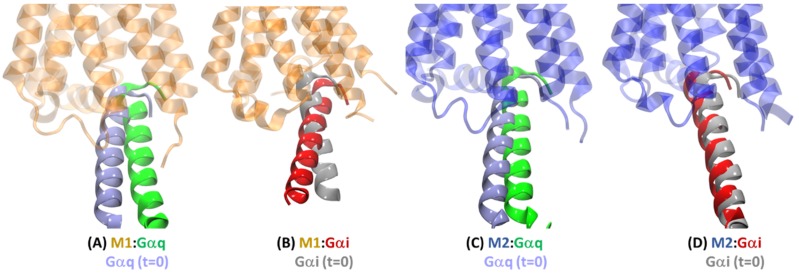
Comparison of α5 helix orientation in the average structures after MD for: (**A**) M1:Gαq, (**B**) M1:Gαi, (**C**) M2:Gαq, and (**D**) M2:Gαi, with those shown in grey at the beginning of those simulations (i.e., at *t* = 0). The rest of the Gα protein is hidden for clarity in all panels.

The average structures from MD simulations show Gαi and Gαq bound through slightly different orientation angles of the α5 helix and Gα protein with respect to the M1 receptor (Figure 6A). However, they are bound to the M2 receptor with very similar orientations (Figure 6B), exhibiting only a relatively slight lateral shift of the α5 helix. As the non-cognate complexes M1:Gαi and M2:Gαq were built using the Go and G11 orientations, respectively, from the cryo-EM structures, it is useful to know any change in orientation caused by the MD simulations. Figure 7 compares the starting orientation of the α5 helix of the Gα complex (*t* = 0) with the α5 helix in the average structure obtained after MD. The Gαq protein in the cognate complex M1:Gαq moved the most (Figure 7A) out of all four complexes. In the other cognate complex M2:Gαi, the Gαi protein moved the least (Figure 7D). The Gα proteins in the non-cognate complexes M1:Gαi and M2:Gαq moved in the intermediate ranges (Figure 7B,C), with the Gαi protein changing its orientation angle in the M1:Gαi complex and the Gαq protein moving slightly laterally in the M2:Gαq complex simulation. These data suggest that the G protein binding cavity in the M1 receptor is broader and is more flexible than that in the M2 receptor. To probe this, the solvent-accessible surface areas (SASA) of the receptor conformations from their two G protein complexes were compared by removing the G proteins. The M1 receptor showed a much bigger difference in SASA for cognate vs non-cognate complexes (~3370 Å^2^) when compared to the M2 receptor (~990 Å^2^), which suggests a more dynamic G-protein-binding pocket in the M1 receptor. In addition, the SASA comparison showed that the non-cognate G protein is held in a tighter space than the cognate G protein for both M1 and M2 receptors, which helps explain the difference in α5 helix orientation that is more evident in the M1 receptor and which might have implications for the G protein activation possible only for the cognate G proteins.

### 2.4. Basis for the G Protein Selectivity of the M1 and M2 Receptors

#### 2.4.1. Thermodynamic Analysis of Cognate and Non-Cognate Complexes

As described in Section 3, the four complexes were relaxed using MD for 2 μs each through four copies of a 0.5 μs simulation for each complex (referred to below as MD1 through MD4). For each saved snapshot for each complex system, the MMPBSA-based free energy of the receptor–G protein binding was calculated and averaged over all snapshots for each of the four MD runs. The results are shown in Table 1.

For the M1 receptor, the cognate complex M1:Gαq had a stronger free energy of binding (−319 kcal/mol) compared to the non-cognate complex M1:Gαi (−270 kcal/mol). Similarly, for the M2 receptor, the cognate complex M2:Gαi had a stronger free energy of binding (−280 kcal/mol) compared to the non-cognate complex M2:Gαq (−248 kcal/mol). The non-cognate complexes were stable during the 2 μs simulation time of the MD simulations, which suggests that both the M1 and M2 receptors are capable of binding to both G proteins; however, the binding affinity of the receptors was weaker for the non-cognate G proteins and stronger for the cognate G proteins (Table 1), providing a thermodynamic basis for the G protein selectivity observed for these receptors. These observations are in line with a previous experimental study on the muscarinic M3 receptor [35], which showed that thermodynamics rather than kinetics plays the determining role in the G protein selectivity shown by the M3 receptor. These findings are also consistent with a recent computational study [34] utilizing the α5 helix of G proteins for receptor–G protein interactions, which showed that the α5 helix of the non-cognate G protein binds more weakly than the α5 helix of the cognate G protein for a given receptor. All these studies in combination with our data imply that the stronger affinity between a receptor and its cognate G protein may be necessary for their coupling and subsequent G protein activation.

#### 2.4.2. Receptor–G Protein Contacts in Cognate and Non-Cognate Complexes

**Figure 8:**
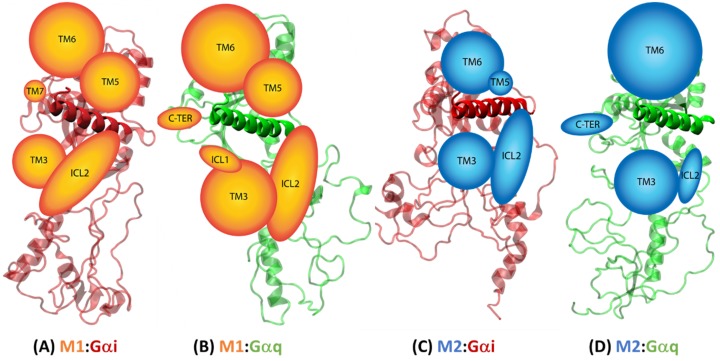
Strong noncovalent interactions between receptors and Gα proteins mapped to individual domains in the receptor for the complexes: (**A**) M1:Gαi, (**B**) M1:Gαq, (**C**) M2:Gαi, and (**D**) M2:Gαq, The size of the receptor domain is proportional to the number of its contacts with the Gα protein (Table S1) and stability of the interaction during the simulation. The structure of the Gα protein is shown but the receptor is only represented by circles for strongly interacting transmembrane (TM) domains and ovals for non-TM domains like intracellular loops (ICLs) and the C-terminus (C-TER).

The snapshots of the four complexes saved during the MD simulations were analyzed for receptor–G protein nonbond contacts using a cutoff distance of 4 Å to identify strong non-covalent interactions (both polar and nonpolar) between the receptor and the Gα protein for each of the complexes. These interactions are summarized in the Supplementary Information (Table S1). The numbers of contacts between a receptor and G protein were analyzed along with the percentage of simulation time during which those contacts were observed. This number was mapped to individual structural domains of the receptors like TM regions or intracellular loops (ICLs) or the C-terminus (C-TER) to identify the strongly interacting receptor domains in the cognate and non-cognate complexes to understand a structural basis for G protein selectivity. These data are shown in Figure 8. The M1 receptor in the cognate complex M1:Gαq (Figure 8B) makes more contacts (using TM3, TM6, ICL2) and stronger contacts than in the non-cognate complex M1:Gαi (Figure 8A). In addition, the M1 receptor uses its C-TER and ICL1 domains to interact (with the Gα) only in the cognate complex and its TM7 domain to interact (with the Gα) only in the non-cognate complex. The M2 receptor uses its ICL2 domain for a stronger interaction in its cognate complex M2:Gαi (Figure 8C) and its TM6 domain for a strong interaction in the non-cognate complex M2:Gαq (Figure 8D). Also, this receptor uses its TM5 domain to interact (with the Gα) only in the cognate complex and its C-TER domain to interact (with the Gα) only in the non-cognate complex. As seen in the previous section, thermodynamically, M2 makes a more strongly binding interaction with Gαi, which is not captured in the visual contact representation portrayed in Figure 8. In addition, the M1 receptor uses TM5 contacts more extensively (Figure 8A,B) compared to the M2 receptor, consistent with the cryo-EM structures [28].

The residues involved in individual interactions between the receptors and Gα proteins are provided in Table S1 along with their relative stability during the 2 μs simulation time. Receptor–G protein contacts specific to the M1 receptor that were found in both M1 complexes but not in the M2 complexes are (ASN422^CTER^-G.H5.22), (THR218^5.68^-G.H5.16), (THR218^5.68^-G.H5.13), (ALA135^IC2^-G.Hns1.3), (ILE211^5.61^-G.H5.25), and (ARG134^IC2^-G.H5.15), where the first residue belongs to the M1 receptor (Ballesteros–Weinstein (BW) residue numbers [38] are shown in superscript; see Supplementary Table S0 for the most conserved BW residues in each TM domain) and the second number refers to a Gα protein residue (common Gα number system [39] shown). The only receptor–G protein contact specific to the M2 receptor that was found in both M2 complexes but not in the M1 complexes is (VAL385^6.33^-G.H5.26). There were interactions found in both Gαi complexes but not in the Gαq complexes: (PRO130^IC2^-ILE345^G.H5.16^), (ALA135^IC2^-ALA31^G.Hns1.2^), (ARG134^IC2^-ASN348^G.H5.19^), and (VAL127^3.54^-LEU349^G.H5.20^). There were no interactions that were only found in the Gαq complexes. LEU131^IC2^ (M1)/LEU129^IC2^ (M2) residue was found buried in the cryo-EM structures [28] and formed contacts in all four complexes. IC2 of M1 and, to a lesser extent, IC2 of M2 made extensive contacts with the G proteins in the MD simulations, similar to in the cryo-EM structures [28]; however, most of these contacts were absent from the non-cognate M2:Gαq complex. The M1/M2 receptor residues 6.33 and 6.36 in TM6 have been implicated in G protein coupling [40,41] but formed interactions with both Gα proteins in the MD structures, suggesting their potentially intricate role in G protein selectivity. In terms of cognate-complex-specific interactions, there were two strong interactions found only in the M2:Gαi complex, (LYS214^5.66^-ASP342^G.H5.13^) and (LYS134^IC2^-GLU28^G.HN.52^), and there were two weaker interactions found only in the M1:Gαq complex, (ARG137^IC2^-ARG37^G.Hns1.2^) and (THR367^6.37^-LEU358^G.H5.25^). Besides these pairs of interactions, there were no other specific interactions found only in cognate receptors, which previous structural bioinformatics studies [17,39] and structural modeling studies [34] had also implied; this supports a bar-code hypothesis for G protein selectivity, where a combination of residue contacts may be responsible for G protein selectivity. The data presented in this study support these findings overall.

#### 2.4.3. Intra-Receptor Contacts in Cognate and Non-Cognate Complexes

In the previous section, some cognate-complex-specific interactions were found between the receptor and G protein; however, they were only strong in the M2:Gαi complex. The findings so far have painted a picture of combinations of receptor–G protein interacting residues that may enable cognate coupling between the receptors and their preferred G proteins. Another source of G protein selectivity may be the intra-receptor interactions that might promote cognate G protein coupling through allosteric effects. GPCRs are known to be very flexible, so there might be receptor-conformation-level signatures that might correlate with cognate G protein coupling. In order to investigate this, we analyzed all intra-receptor interacting residues for all four complexes along the MD trajectories (see Table S2). The data show many intra-receptor interactions present in all four complexes. This is not surprising, given the high sequence similarity between the M1 and M2 receptors. The data also show many M1 receptor-specific and M2 receptor-specific interactions found in both of their respective complexes. The cryo-EM structure of the M2 complex showed residue 5.62 of M2 interacting with residue 6.34, which we found in both the cognate and non-cognate M2 complexes and in the cognate M1 complex. This residue 5.62 has been implicated in Gq selectivity [42], so the explanation of its exact role may be more nuanced based on the cryo-EM structure and our MD simulations. We did find a conserved contact that is only present in the cognate complexes: M1:Gαq (SER184^5.34^-THR390^6.59^) and M2:Gαi (SER182^5.34^-THR411^6.59^). This contact, being in the extracellular-facing half of the TM domain, is an excellent choice to mutate in future studies to tease out its mechanistic role in G protein selectivity as it is present far away from the G-protein-binding receptor interface.

## 3. Materials and Methods

### 3.1. Receptor–G Protein Complex Structure Preparation

The receptor-bound conformations of Gαi and Gαq proteins were built through the SWISS-MODEL webserver [43] based on the Go and G11 protein conformations from the M2:Go (PDB: 6OIK) and M1:G11 (PDB: 6OIJ) complexes, respectively [28]. The G-protein-bound receptor conformations of the M1 and M2 receptors were also built through the SWISS-MODEL webserver based on the M1 and M2 receptor conformations from the abovementioned M1:G11 and M2:Go complexes [28]. The Visual Molecular Dynamics (VMD) software program [44] was used to create the two cognate receptor–G protein complexes (M1:Gαq and M2:Gαi) and two non-cognate complexes (M1:Gαi and M2:Gαq) based on the M1:G11 and M2:Go complex structures. The minor residue–sidechain steric clashes in the non-cognate complexes (M1:Gαi and M2:Gαq) were resolved using the SCWRL4 method [45]. The coordinates of the agonist ligand iperoxo from the M1:G11 and M2:Go complexes [28] were used in the M1- and M2-based complexes, respectively.

Each of the four complexes was inserted into a POPC (phosphatidylcholine) lipid bilayer of dimensions 105 Å × 105 Å and a combined water layer of 95 Å above/below the lipid using the Membrane Builder module of CHARMM-GUI [46]. The molecular systems were neutralized through the addition of Na^+^ or Cl^–^ ions. The number of atoms in the four systems ranged between 145,000 and 155,000. The Charmm36 force field [47] was used for the proteins, lipids, and ions. The TIP3P force field [48] was used for the water molecules. The force field for the ligand iperoxo was parametrized through the ligand reader/modeler module of CHARMM-GUI [49]. The protein topology files for implicit-solvent-energy-based MMPBSA analysis were acquired through the Implicit Solvent Modeler in CHARMM-GUI [46].

### 3.2. Molecular Dynamics

Each of the four receptor–G protein complexes was relaxed in the lipid bilayer environment through a series of energy minimization and equilibration steps implemented in the Amber 16 simulation program [50]. The equilibration was done using NPT (constant Number of atoms, constant Pressure, and constant Temperature) molecular dynamics (MD), in which a Berendsen barostat and thermostat were used to maintain a constant pressure of 1 atm and a constant temperature of 310 K, respectively, to mimic physiological conditions. Periodic boundary conditions were used and the electrostatics were handled by the particle mesh Ewald summation method. The non-bond interaction cutoff was 8 Å and the simulation time step was 2 fs. Here are the six relaxation steps:

1. The lipids and waters were energy minimized for 2000 steps using a sequence of 1000 steepest descent steps and 1000 conjugate gradient steps while keeping the protein and ligand fixed. This removed any protein–solvent steric clashes and prepared the solvent for the next step.
2. The lipids and waters were then relaxed using NPT equilibration MD for a simulation time of 250 ps while keeping the protein and ligand fixed. This allowed for any artificial air bubbles at the protein–solvent interface to be filled in by the solvent.
3. The whole molecular system was then energy minimized for 2000 steps (like in Step 1) to allow the protein atoms to adjust to the relaxed solvent.
4. The whole molecular system was then slowly heated from 0 K to 310 K at 1 atm pressure over a simulation time of 100 ps.
5. The whole system was then relaxed using NPT equilibration at 310 K and 1 atm until the system density was stabilized, which typically occurred within 250 ps.
6. Finally, the whole system was equilibrated for 0.5 µs. Simulation snapshots were saved every 10 ps for subsequent structural and thermodynamic analysis.

This six-step procedure was carried out for each of the four complex systems, and the last step was replicated four times with reinitialized velocities for 0.5 μs each for a total of 2 µs of MD simulation per system.

### 3.3. Trajectory Analysis

The RMSD Trajectory Tool plugin in VMD [44] was used to extract the average receptor–G protein complex structures from the simulation. The sequences and coordinates of the average structures were aligned using the Multiseq plugin in VMD. The RMSD values of the aligned protein regions were calculated using the RMSD Tool plugin in VMD. The hydrogen bonds were analyzed using the Hbond plugin in VMD. Polar and non-polar interactions were analyzed using the native contacts script with a non-bond interaction cutoff distance of 4 Å in the CPPTRAJ utility [51] of the AMBER simulation package [50].

### 3.4. Binding Free Energy Analysis

**Figure 9:**
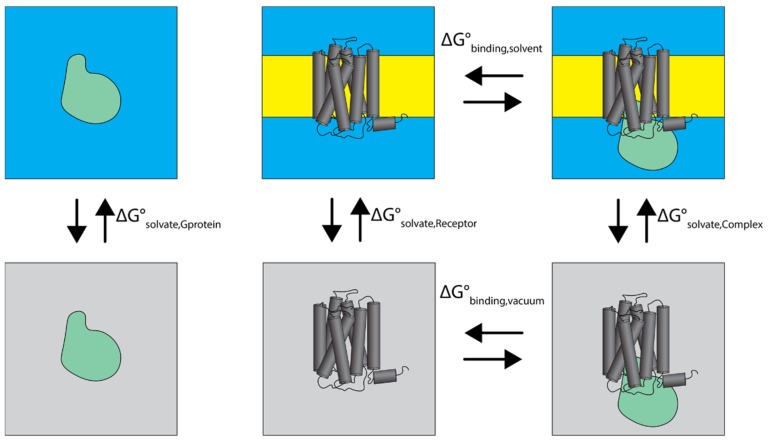
The MMPBSA thermodynamic cycle of the G protein (green blob) in aqueous phase (blue) binding to the G-protein-coupled receptor (GPCR) (dark gray) embedded in the membrane (yellow). The lower three panels refer to vacuum conditions, shown in light gray. The free energy change for different transitions is shown as ΔG°.

Molecular mechanics/Poisson–Boltzmann surface area (MMPBSA) calculations [36,37] were used to calculate the change in the free energy of binding of the G protein to the receptor. The binding free energy for the receptor–G protein complex was calculated as the difference between the complex free energy and the sum of the receptor and G protein free energies as depicted by the thermodynamic cycle of receptor–G protein binding shown in Figure 9. The MMPBSA calculations were carried out either in implicit water (for the G protein) or in an implicit membrane–water system (for the receptor and the receptor–G protein complex). The equation governing the free energy change upon binding shown in Figure 9 is

(1) $$\Delta G_{binding,solvent}^{o}=\Delta G_{binding,vacuum}^{o}+\Delta G_{solvate,Complex}^{o}-\Delta G_{solvate,Gprotein}^{o}-\Delta G_{solvate,Receptor}^{o}$$

where each solvation free energy term has two components:

(2) $$\Delta G_{solvate}^{o}=\Delta G_{electrostatic}^{o}+\Delta G_{nonpolar}^{o}$$

For the MMPBSA calculations, a uniform single dielectric membrane model was chosen with the membrane dielectric constant set to 7. The average membrane thickness was calculated for each trajectory between 30 and 45 Å. Other Poisson–Boltzmann (PB) settings included the use of periodic boundary conditions, the atom-based cutoff distance for pairwise charge-based interactions set to 7.0 Å, the atom-based cutoff distance for van der Waals interactions set to 99.0 Å, the ionic strength set to 150 mM, and the ratio between the dimension of the finite-difference grid and that of the solute box set to 1.5. The total electrostatic energies and forces were calculated using the particle–particle particle–mesh (P3M) procedure, the electrostatic focusing was disabled, Bondi radii from the parameter topology file were enabled, and the pore searching algorithm was disabled. All other PB parameters were set to their default values in MMPBSA.py from Amber 17 [50]. The free energy of receptor–G protein binding was calculated for each saved MD snapshot and then averaged over all snapshots from a given simulation.

## 4. Conclusions

This study used the recent cryo-EM structures of the M1:G11 and M2:Go complexes to build the structures of cognate (M1:Gαq/M2:Gαi) and non-cognate (M1:Gαi/M2:Gαq) complexes and relaxed the four complexes in the membrane environment using MD simulations. A structural analysis of both the cognate and non-cognate complexes combined with thermodynamic studies on the binding free energies showed that the M1 and M2 receptors can couple to both Gα proteins; however, the cognate partners display stronger binding affinity than the non-cognate partners. This is consistent with previous experimental studies on the M3 receptor and computational studies on receptor–α5 helix interactions, where thermodynamics was shown to play a critical role in G protein selectivity. Detailed analysis of the receptor–G protein interactions found in cognate and non-cognate complexes showed the conformational malleability of the intracellular-facing domains of receptors that enables them to interact with multiple G proteins. This conformational flexibility could be used to design receptor mutants with altered G protein specificities, as has been demonstrated before [34,40,41]. This study also identified a TM5–TM6 interaction away from the G-protein-binding region of the receptors that is present only in the cognate complexes. This highlights the unique signatures that might be distributed throughout the receptor and which can affect G protein coupling allosterically. The non-cognate complexes are harder to characterize experimentally but provide much-needed insight into the G protein preferences displayed by GPCRs in cells. So, these simulations fill a structural gap in our understanding of receptor–G protein interactions and also provide a mechanistic framework to think about the G protein selectivity of GPCRs and the mutagenesis studies that associate certain receptors or G protein residues with G protein coupling or selectivity. With respect to the latter, we have shown in this study that the explanations of the roles of some residues in G protein selectivity may not be so straightforward.

## Figures and Tables

**Table 1:** The molecular mechanics/Poisson–Boltzmann surface area (MMPBSA)-based free energy of binding of the Gα protein to the receptor. Energies are shown for each of the four MD runs for each of the four complexes, along with the average free energy of binding in **bold** for each complex in kcal/mol.

R:G Binding Free Energy (kcal/mol)	M1:Gαi	M1:Gαq	M2:Gαi	M2:Gαq
**MD1**	−318	−233	−259	−272
**MD2**	−252	−353	−285	−184
**MD3**	−221	−327	−325	−281
**MD4**	−228	−363	−253	−253
**Average**	**−270**	**−319**	**−280**	**−248**
**StdDev**	42	59	33	44

## Supplementary Materials

Supplementary materials can be found at https://www.mdpi.com/1422-0067/20/21/5290/s1.

## Abbreviations

GPCR	G-protein-coupled receptor
MD	Molecular dynamics
MMPBSA	Molecular mechanics/Poisson–Boltzmann surface area
VMD	Visual molecular dynamics
RMSD	Root-mean-squared deviation
