# Supplementary material for: Understanding G Protein Selectivity of Muscarinic Acetylcholine Receptors Using Computational Methods
Source: Int J Mol Sci. 2019 Oct 24;20(21):5290. doi: 10.3390/ijms20215290 (PMC6862617; doi:10.3390/ijms20215290)
Supplement: Supplementary file 1 [file ijms-20-05290-s001.pdf]

Understanding G protein selectivity of muscarinic acetylcholine receptors using computational methods

Luis Jaimes Santiago and Ravinder Abrol*

Department of Chemistry and Biochemistry, California State University, Northridge, CA 91330, USA

* Correspondence: abrol@csun.edu; Tel.: +1-818-677-5454 (R.A.)

Supplementary Information

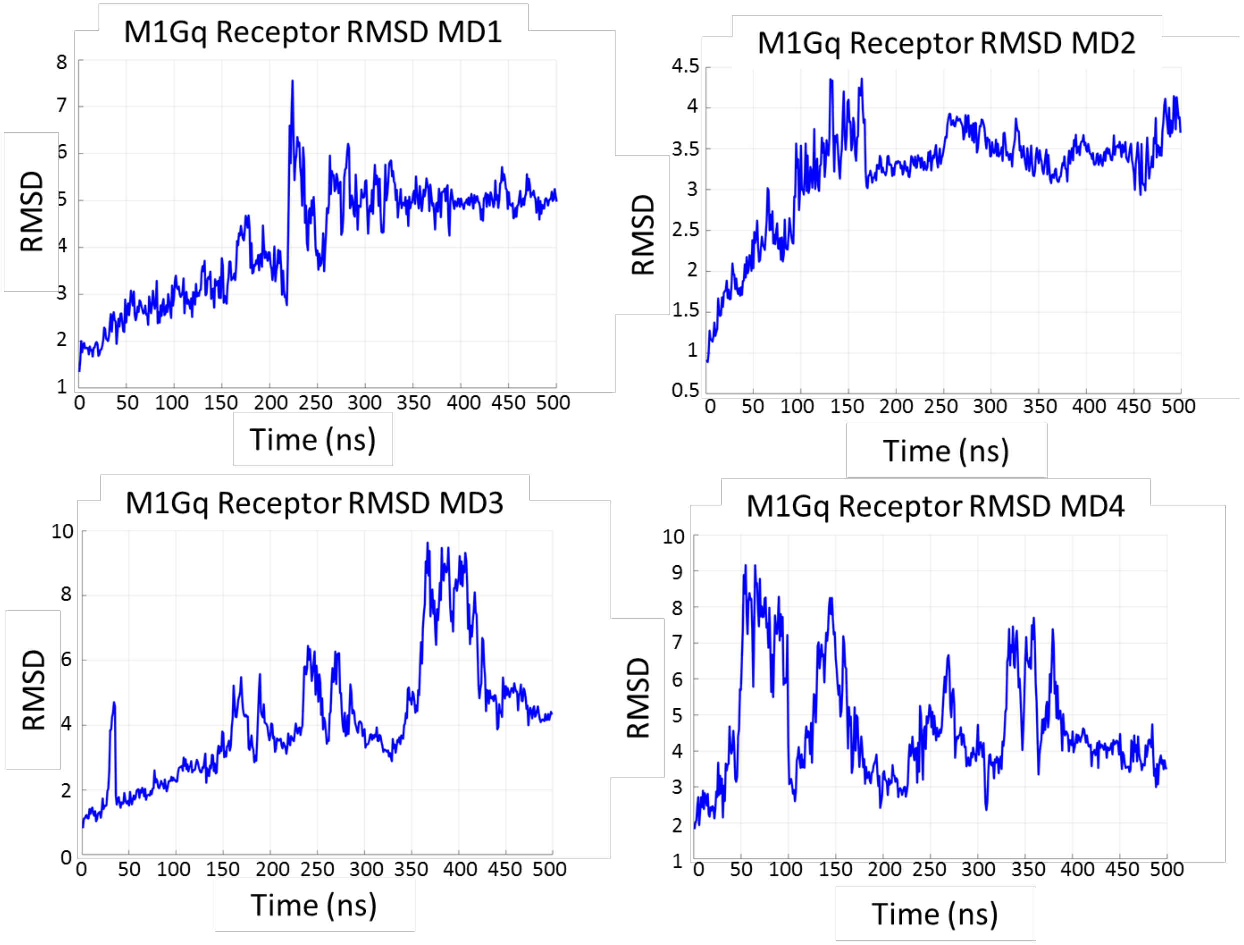

Figure S1: RMSD of the M1 Receptor in M1:Gαq complex for 4x500ns MD simulations.

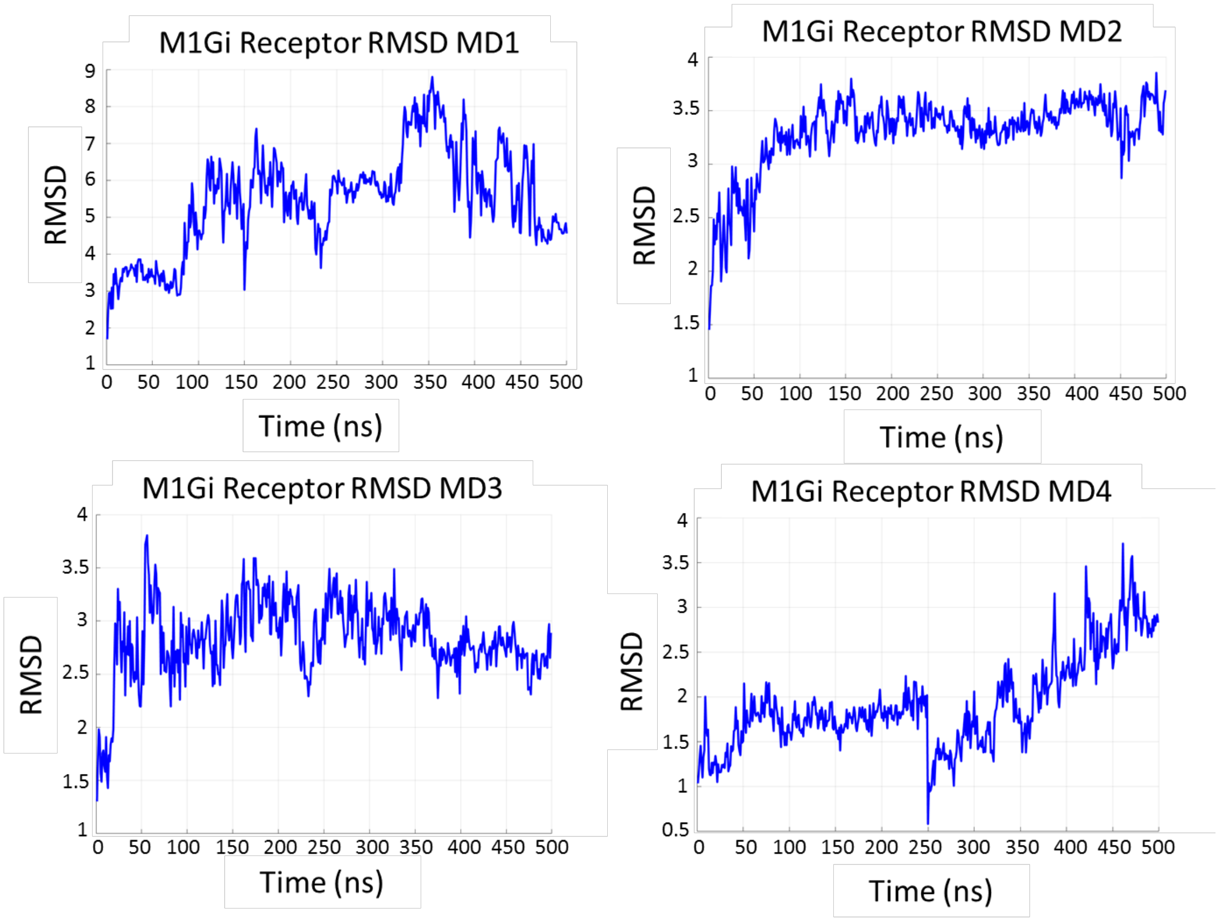

Figure S2: RMSD of the M1 Receptor in M1:Gαi complex for 4x500ns MD simulations.

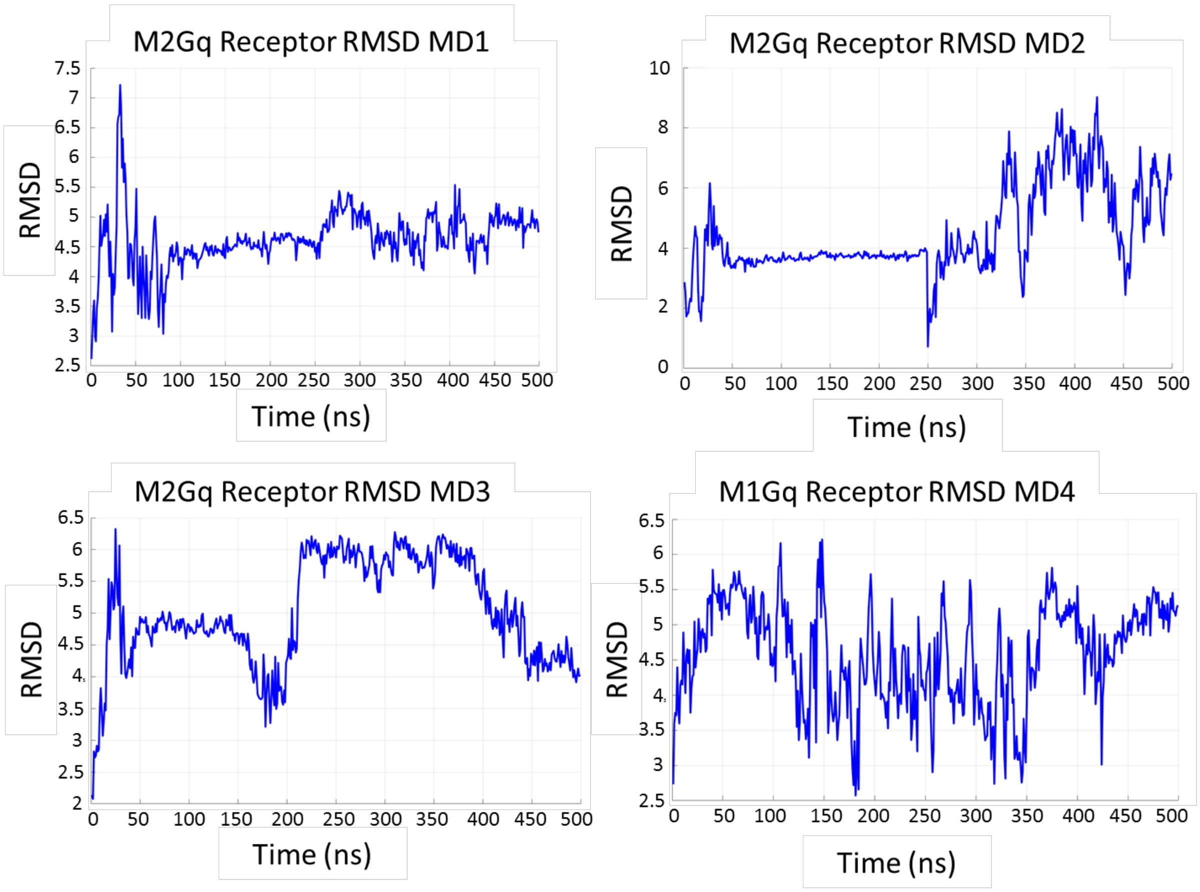

Figure S3: RMSD of the M2 Receptor in M2:Gαq complex for 4x500ns MD simulations.

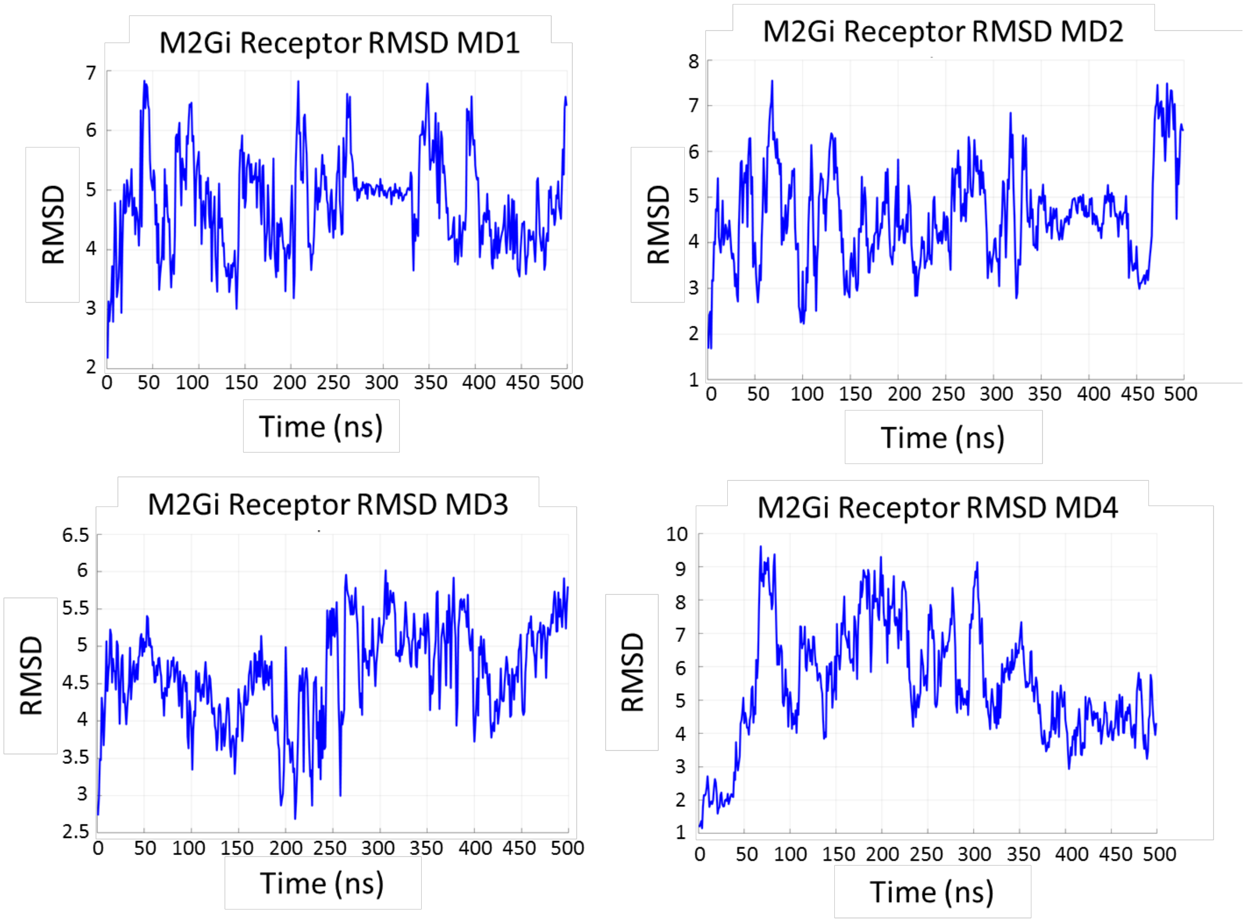

Figure S4: RMSD of the M2 Receptor in M2:Gαi complex for 4x500ns MD simulations.

Domains	M1R	M2R
TM1	23-52	21-50
TM2	59-87	57-85
TM3	95-128	93-126
TM4	139-163	137-161
TM5	184-217	182-215
TM6	358-390	380-412
TM7	395-421	417-443
1.50 Residue	N43	N41
2.50 Residue	D71	D69
3.50 Residue	R123	R121
4.50 Residue	W150	W148
5.50 Residue	P200	P198
6.50 Residue	P380	P402
7.50 Residue	P415	P437

Table S0: The residue numbers for the TM domains for the two receptors are shown along with the most conserved residue in each TM domain that defines the Ballesteros-Weinstein numbering scheme, useful for comparing different receptors [1].

- Hydrogen bonds
- Key M2 interaction
- Key M1 interaction
- Residues in M1(AALS)/M2(VTIL) motif
- Mentioned in M1/M2 cryo-EM paper
- Common across all systems

M1:Gq			M2:Gq			M1:Gi			M2:Gi		
BW	GN	Pop	BW	GN	Pop	BW	GN	Pop	BW	GN	Pop
ARG123$^{3.50}$	TYR356$^{G.H5.23}$	99%	ARG121$^{3.50}$	TYR356$^{G.H5.23}$	94%	ARG123$^{3.50}$	CYS352$^{G.H5.23}$	73%	ARG121$^{3.50}$	CYS352$^{G.H5.23}$	87%
SER126$^{3.53}$	ASN352$^{G.H5.19}$	77%	CYS124$^{3.53}$	ASN352$^{G.H5.19}$	84%	SER126$^{3.53}$	ASN348$^{G.H5.19}$	89%	CYS124$^{3.53}$	ASN348$^{G.H5.19}$	96%
LYS362$^{6.32}$	VAL359$^{G.H5.26}$	92%	LYS384$^{6.32}$	VAL359$^{G.H5.26}$	81%	LYS362$^{6.32}$	PHE355$^{G.H5.26}$	91%	LYS384$^{6.32}$	PHE355$^{G.H5.26}$	68%
ALA363$^{6.33}$	LEU358$^{G.H5.25}$	89%	VAL385$^{6.33}$	LEU358$^{G.H5.25}$	86%	ALA363$^{6.33}$	LEU354$^{G.H5.25}$	76%	VAL385$^{6.33}$	LEU354$^{G.H5.25}$	71%
VAL127$^{3.54}$	LEU349$^{G.H5.16}$	83%	VAL125$^{3.54}$	LEU349$^{G.H5.16}$	76%	VAL127$^{3.54}$	ILE345$^{G.H5.16}$	76%	VAL125$^{3.54}$	ILE345$^{G.H5.16}$	47%
THR366$^{6.36}$	LEU358$^{G.H5.25}$	60%	THR388$^{6.36}$	LEU358$^{G.H5.25}$	79%	THR366$^{6.36}$	LEU354$^{G.H5.25}$	53%	THR388$^{6.36}$	LEU354$^{G.H5.25}$	70%
CYS421$^{7.56}$	ASN357$^{G.H5.24}$	78%	CYS443$^{7.56}$	ASN357$^{G.H5.24}$	77%	CYS421$^{7.56}$	GLY353$^{G.H5.24}$	38%	CYS443$^{7.56}$	GLY353$^{G.H5.24}$	48%
SER126$^{3.53}$	TYR356$^{G.H5.23}$	99%	CYS124$^{3.53}$	TYR356$^{G.H5.23}$	98%			0%	CYS124$^{3.53}$	CYS352$^{G.H5.23}$	42%
PRO130^{IC2}	ASN348$^{G.H5.15}$	32%	PRO128^{IC2}	ASN348$^{G.H5.15}$	85%	PRO130^{IC2}	ILE344$^{G.H5.15}$	68%	PRO128^{IC2}	ILE344$^{G.H5.15}$	46%
LYS362$^{6.32}$	LEU358$^{G.H5.25}$	61%	LYS384$^{6.32}$	LEU358$^{G.H5.25}$	81%	LYS362$^{6.32}$	LEU354$^{G.H5.25}$	44%	LYS384$^{6.32}$	LEU354$^{G.H5.25}$	32%
LEU131^{IC2}	VAL199$^{G.S3.1}$	35%	LEU129^{IC2}	VAL199$^{G.S3.1}$	58%	LEU131^{IC2}	LEU195$^{G.S3.1}$	76%	LEU129^{IC2}	LEU195$^{G.S3.1}$	39%
PRO130^{IC2}	ASN352$^{G.H5.19}$	29%	PRO128^{IC2}	ASN352$^{G.H5.19}$	53%	PRO130^{IC2}	ASN348$^{G.H5.19}$	56%	PRO128^{IC2}	ASN348$^{G.H5.19}$	64%
LEU131^{IC2}	LYS345$^{G.H5.12}$	54%	LEU129^{IC2}	LYS345$^{G.H5.12}$	42%	LEU131^{IC2}	THR341$^{G.H5.12}$	77%	LEU129^{IC2}	THR341$^{G.H5.12}$	27%
THR215$^{5.65}$	LEU353$^{G.H5.20}$	63%	SER213$^{5.65}$	LEU353$^{G.H5.20}$	50%	THR215$^{5.65}$	LEU349$^{G.H5.20}$	74%			0%
THR366$^{6.36}$	ASN357$^{G.H5.24}$	71%	THR388$^{6.36}$	ASN357$^{G.H5.24}$	52%	THR366$^{6.36}$	GLY353$^{G.H5.24}$	60%			0%
LEU367$^{6.37}$	LEU358$^{G.H5.25}$	27%	ILE389$^{6.37}$	LEU358$^{G.H5.25}$	49%	LEU367$^{6.37}$	LEU354$^{G.H5.25}$	52%	ILE389$^{6.37}$	LEU354$^{G.H5.25}$	51%
LEU131^{IC2}	PHE341$^{G.H5.8}$	36%	LEU129^{IC2}	PHE341$^{G.H5.8}$	52%	LEU131^{IC2}	PHE337$^{G.H5.8}$	48%	LEU129^{IC2}	PHE337$^{G.H5.8}$	25%
LEU131^{IC2}	ASN348$^{G.H5.15}$	50%			0%	LEU131^{IC2}	ILE344$^{G.H5.15}$	57%	LEU129^{IC2}	ILE344$^{G.H5.15}$	54%
ASN422^{CTER}	GLU355$^{G.H5.22}$	81%			0%	ASN422^{CTER}	ASP351$^{G.H5.22}$	72%			0%
PRO130^{IC2}	LEU349$^{G.H5.16}$	25%			0%	PRO130^{IC2}	ILE345$^{G.H5.16}$	74%	PRO128^{IC2}	ILE345$^{G.H5.16}$	47%
ALA135^{IC2}	ARG37$^{G.Hns1.2}$	20%			0%	ALA135^{IC2}	ALA31$^{G.Hns1.2}$	56%	VAL133^{IC2}	ALA31$^{G.Hns1.2}$	50%
ARG134^{IC2}	ASN352$^{G.H5.19}$	64%			0%	ARG134^{IC2}	ASN348$^{G.H5.19}$	24%	PRO132^{IC2}	ASN348$^{G.H5.19}$	34%
VAL127$^{3.54}$	LEU353$^{G.H5.20}$	32%			0%	VAL127$^{3.54}$	LEU349$^{G.H5.20}$	35%	VAL125$^{3.54}$	LEU349$^{G.H5.20}$	53%
THR218$^{5.68}$	LEU349$^{G.H5.16}$	37%			0%	THR218$^{5.68}$	ILE345$^{G.H5.16}$	78%			0%
THR218$^{5.68}$	ASP346$^{G.H5.13}$	40%			0%	THR218$^{5.68}$	ASP342$^{G.H5.13}$	73%			0%
ALA135^{IC2}	ARG38$^{G.Hns1.3}$	55%			0%	ALA135^{IC2}	ARG32$^{G.Hns1.3}$	54%			0%
ILE211$^{5.61}$	LEU358$^{G.H5.25}$	38%			0%	ILE211$^{5.61}$	LEU354$^{G.H5.25}$	59%			0%
		0%	ARG381$^{6.29}$	GLN350$^{G.H5.17}$	86%			0%			0%
ARG134^{IC2}	ILE348$^{G.H5.15}$	29%			0%	ARG134^{IC2}	ILE344$^{G.H5.15}$	53%			0%
		0%	VAL385$^{6.33}$	VAL359$^{G.H5.26}$	55%			0%	VAL385$^{6.33}$	PHE355$^{G.H5.26}$	26%
		0%	ASN444^{CTER}	ASN357$^{G.H5.24}$	76%			0%			0%
		0%	ASN444^{CTER}	GLU355$^{G.H5.22}$	75%			0%			0%
		0%			0%			0%	LYS214$^{5.66}$	ASP342$^{G.H5.13}$	74%
		0%			0%			0%	LYS134^{IC2}	GLU28$^{G.HN.52}$	73%
		0%	ARG381$^{6.29}$	ASP346$^{G.H5.13}$	73%			0%			0%
		0%			0%	ARG134^{IC2}	ALA31$^{G.Hns1.2}$	57%			0%
ARG137^{IC2}	ARG37$^{G.Hns1.2}$	35%									
THR367$^{6.37}$	LEU358$^{G.H5.25}$	27%									

Table S1: Receptor—G-protein noncovalent interactions in the four receptor—G-protein complexes. Residue names and numbers are followed by their Ballesteros-Weinstein numbers for GPCRs [1], common Gα numbering system for Gα protein residues [2] and % of the 2 μs simulation time that those interactions are observed.

Conserved | M1 Unique | From Literature | M2 Unique

M1 Systems						M1:Gq	M1:Gi	M2 systems						M2:Gq	M2:Gi
ASP	71	2.50	SER	411	7.46	84%	94%	ASP	69	2.50	SER	433	7.46	91%	94%
GLN	110	3.37	TRP	157	4.57	93%	84%	ASN	108	3.37	TRP	155	4.57	89%	72%
THR	39	1.46	THR	412	7.47	87%	88%	THR	37	1.46	THR	434	7.47	78%	75%
GLN	110	3.37	SER	153	4.53	80%	77%	ASN	108	3.37	SER	151	4.53	82%	88%
SER	78	2.57	ASP	105	3.32	79%	78%	SER	76	2.57	ASP	103	3.32	82%	86%
ASN	43	1.50	ALA	68	2.47	73%	81%	ASN	41	1.50	ALA	66	2.47	74%	92%
TYR	106	3.33	TRP	157	4.57	84%	76%	TYR	104	3.33	TRP	155	4.57	82%	76%
SER	36	1.43	ASN	80	2.59	68%	71%	SER	34	1.43	ASN	78	2.59	80%	97%
PHE	125	3.52	TYR	133	ICL2	95%	55%	PHE	123	3.52	TYR	131	ICL2	85%	81%
TYR	85	2.64	CYS	177	ECL2	64%	74%	TYR	83	2.64	CYS	176	ECL2	92%	85%
PHE	121	3.48	LEU	207	5.57	71%	79%	PHE	119	3.48	LEU	205	5.57	80%	79%
ALA	111	3.38	SER	153	4.53	77%	77%	ALA	109	3.38	SER	151	4.53	78%	77%
SER	66	2.45	ASN	115	3.42	74%	82%	SER	64	2.45	ASN	113	3.42	70%	82%
ASN	43	1.50	ASP	71	2.50	79%	74%	ASN	41	1.50	ASP	69	2.50	82%	71%
CYS	98	3.25	CYS	178	ECL2	86%	79%	CYS	96	3.25	CYS	176	ECL2	70%	68%
ALA	111	3.38	TRP	150	4.50	64%	73%	ALA	109	3.38	TRP	148	4.50	84%	81%
ASP	99	3.26	ARG	171	ECL2	83%	58%	ASP	97	3.26	ARG	169	ECL2	85%	72%
ASP	122	3.49	TYR	133	ICL2	66%	89%	ASP	120	3.49	TYR	131	ICL2	78%	66%
**TYR**	**208**	**5.58**	**LEU**	**367**	**6.37**	**71%**	**69%**	**TYR**	**206**	**5.58**	**ILE**	**389**	**6.37**	**76%**	**81%**
VAL	107	3.34	SER	153	4.53	64%	74%	VAL	105	3.34	SER	151	4.53	76%	82%
LEU	81	2.60	TRP	101	3.28	65%	80%	LEU	79	2.60	TRP	99	3.28	74%	78%
TYR	106	3.33	TYR	381	6.51	57%	86%	TYR	104	3.33	TYR	403	6.51	68%	82%
TRP	377	6.47	ASN	410	7.45	80%	61%	THR	399	6.47	ASN	432	7.45	66%	83%
TYR	106	3.33	ILE	180	ECL2	77%	81%	TYR	104	3.33	ILE	178	ECL2	60%	70%
SER	78	2.57	TRP	101	3.28	71%	52%	SER	76	2.57	TRP	99	3.28	83%	77%
SER	36	1.43	GLY	75	2.54	56%	78%	SER	34	1.43	GLY	73	2.54	69%	79%
PHE	197	5.47	TRP	379	6.48	80%	65%	PHE	195	5.47	TRP	400	6.48	70%	64%
LEU	183	ECL2	THR	189	5.39	56%	74%	PHE	181	ECL2	THR	187	5.39	71%	79%
TYR	124	3.51	LEU	207	5.57	79%	67%	TYR	122	3.51	LEU	205	5.57	61%	71%
TYR	124	3.51	ARG	210	5.60	71%	65%	TYR	122	3.51	HSD	208	5.60	72%	70%
ASN	43	1.50	PRO	415	7.50	73%	75%	ASN	41	1.50	PRO	437	7.50	67%	60%
PHE	63	2.42	LEU	118	3.45	67%	61%	PHE	61	2.42	ILE	116	3.45	71%	75%
THR	189	5.39	THR	389	6.59	63%	74%	THR	187	5.39	THR	411	6.59	72%	61%
TRP	91	ECL1	TRP	101	3.28	56%	56%	TRP	89	ECL1	TRP	99	3.28	80%	75%
THR	32	1.39	THR	83	2.62	74%	72%	ALA	30	1.39	THR	81	2.62	62%	57%
LEU	56	ICL1	ASN	61	2.40	82%	76%	LEU	54	ICL1	ASN	59	2.40	87%	19%
PHE	197	5.47	ASN	382	6.52	52%	73%	PHE	195	5.47	ASN	404	6.52	60%	78%
SER	126	3.53	TYR	133	ICL2	68%	55%	CYS	124	3.53	TYR	131	ICL2	66%	63%
ASN	60	2.39	ASP	122	3.49	73%	65%	ASN	58	2.39	ASP	120	3.49	70%	43%
TYR	124	3.51	ILE	211	5.61	64%	69%	TYR	122	3.51	ILE	209	5.61	67%	51%
ALA	160	4.60	PHE	182	ECL2	73%	65%	ALA	158	4.60	PHE	180	ECL2	58%	54%
SER	49	1.56	THR	428	Cter	72%	76%	SER	47	1.56	THR	450	Cter	61%	40%
TYR	82	2.61	GLU	401	7.36	60%	54%	TYR	80	2.61	THR	423	7.36	70%	63%
TRP	164	ECL2	GLN	181	ECL2	86%	0%	TRP	162	ECL2	GLN	179	ECL2	77%	83%
PHE	50	1.57	LYS	57	ICl1	77%	87%	ILE	48	1.57	GLN	55	ICl1	74%	5%
PHE	50	1.57	LEU	65	2.44	59%	69%	ILE	48	1.57	PHE	63	2.44	47%	64%
SER	120	3.47	LEU	207	5.57	54%	66%	SER	118	3.47	LEU	205	5.57	66%	50%
ASN	43	1.50	LEU	72	2.51	57%	73%	ASN	41	1.50	LEU	70	2.51	48%	51%
PHE	50	1.57	LEU	56	ICL1	63%	66%	ILE	48	1.57	LEU	54	ICL1	66%	34%
TYR	85	2.64	GLN	176	ECL2	0%	65%	TYR	83	2.64	GLU	175	ECL2	80%	79%
TRP	91	ECL1	CYS	98	3.25	56%	0%	TRP	89	ECL1	CYS	96	3.25	85%	77%
TYR	85	2.64	HSD	90	ECL1	79%	66%	TYR	83	2.64	TRP	89	ECL1	62%	73%
TYR	82	2.61	TYR	408	7.43	68%	50%	TYR	80	2.61	TYR	430	7.43	33%	50%
MET	384	6.54	TRP	400	7.35	57%	59%	MET	406	6.54	TRP	422	7.35	76%	63%

Table S2 contd…

Conserved in Cognate Complexes Only | M1 Unique | Conserved | M2 Unique

M1 Systems						M1:Gq	M1:Gi	M2 systems						M2:Gq	M2:Gi
TYR	212	5.62	ALA	364	6.34	58%	0%	SER	210	5.62	THR	386	6.34	74%	58%
PRO	186	5.36	THR	390	6.59	60%	69%	ALA	184	5.36	THR	411	6.59	0%	59%
ALA	70	2.49	ALA	108	3.35	50%	0%	ALA	68	2.49	VAL	106	3.35	68%	70%
GLY	42	1.49	PRO	415	7.50	0%	58%	GLY	40	1.49	PRO	437	7.50	56%	71%
SER	49	1.56	PHE	425	Cter	73%	0%	SER	47	1.56	PHE	447	Cter	46%	65%
TYR	82	2.61	TYR	404	7.39	0%	52%	TYR	80	2.61	TYR	426	7.39	69%	61%
LYS	51	1.58	LYS	57	ICL1	58%	65%	LYS	49	1.58	GLN	55	ICL1	54%	5%
								ASP	103	3.32	TYR	430	7.43	87%	88%
SER	49	1.56	LEU	56	ICL1	50%	57%	SER	47	1.56	LEU	54	ICL1	58%	0%
								ALA	441	7.54	PHE	447	Cter	84%	79%
								ASP	97	3.26	ARG	169	ECL2	85%	72%
ASN	43	1.50	SER	411	7.46	0%	58%	ASN	41	1.50	SER	433	7.46	46%	52%
								SER	76	2.57	TYR	430	7.43	73%	79%
								GLN	163	ECL2	ARG	169	ECL2	64%	88%
SER	36	1.43	MET	79	2.58	77%	73%								
LEU	419	7.54	ARG	426	Cter	0%	61%	ALA	441	7.54	LYS	448	Cter	24%	61%
								TRP	400	6.48	CYS	429	7.42	80%	65%
								THR	399	6.47	LEU	428	7.41	75%	66%
								MET	45	1.54	PHE	63	2.44	80%	59%
								ASP	120	3.49	ARG	135	ICL2	80%	58%
GLN	165	ecl2	ARG	171	ecl2	65%	70%								
								GLN	163	ecl2	VAL	168	ecl2	63%	70%
SER	184	5.34	THR	390	6.59	64%	0%	SER	182	5.34	THR	411	6.59	0%	63%
								LEU	442	7.55	LYS	448	Cter	67%	55%
								MET	45	1.54	ALA	66	2.47	59%	61%
ASN	60	2.39	ARG	136	ICL2	0%	53%	ASN	58	2.39	ARG	135	ICL2	57%	9%
								MET	45	1.54	CYS	67	2.48	57%	61%
								ILE	48	1.57	ASN	59	2.40	46%	67%
								TYR	80	2.61	TRP	99	3.28	51%	56%
								TRP	400	6.48	ASN	432	7.45	44%	62%
								SER	107	3.36	TRP	400	6.48	45%	59%
								TYR	80	2.61	TRP	427	7.40	49%	54%
								THR	187	5.39	VAL	407	6.55	49%	51%
								ASN	41	1.50	LEU	70	2.51	48%	51%
								ILE	48	1.57	GLN	55	ICL1	74%	5%
								VAL	44	1.53	ALA	441	7.54	51%	27%
								GLN	55	ICL1	TYR	60	2.41	55%	20%
								SER	16	Nter	ILE	86	ECL1	22%	50%

Table S2: Intra-receptor noncovalent interactions in the four receptor—G-protein complexes. Residue names and numbers are followed by their Ballesteros-Weinstein numbers [1] and % of 2 μs simulation time that those interactions are observed.

References

1. Ballesteros, J.A.; Weinstein, H. Integrated methods for the construction of threedimensional models and computational probing of structure-function relations in G protein-coupled receptors. In *Methods in Neurosciences*, Sealfon, S.C., Ed. Academic: 1995; Vol. 25, pp. 366-428.
2. Flock, T.; Ravarani, C.N.J.; Sun, D.; Venkatakrishnan, A.J.; Kayikci, M.; Tate, C.G.; Veprintsev, D.B.; Babu, M.M. Universal allosteric mechanism for Galpha activation by GPCRs. *Nature* **2015**, *524*, 173-179, doi:10.1038/nature14663.
